# Post-adolescent developmental changes in cortical complexity

**DOI:** 10.1186/1744-9081-10-44

**Published:** 2014-11-27

**Authors:** Anca-Larisa Sandu, Edouard Izard, Karsten Specht, Harald Beneventi, Arvid Lundervold, Martin Ystad

**Affiliations:** Aberdeen Biomedical Imaging Centre, Lilian Sutton Building, University of Aberdeen, Foresterhill, AB25 2ZD Aberdeen, UK; Department of Biological and Medical Psychology, University of Bergen, Jonas Lies vei 91, N-5009 Bergen, Norway; Department of Biomedicine, Jonas Lies vei 91, N-5009 Bergen, Norway; Department of Medical Engineering, Haukeland University Hospital, Bergen, Norway; Department of Radiology, Haukeland University Hospital, Bergen, Norway

**Keywords:** Grey matter, Fractal dimension, Development, Dimorphism, Magnetic resonance imaging

## Abstract

**Background:**

Post-adolescence is known to be a period of general maturation and development in the human brain. In brain imaging, volumetric and morphologic cortical grey-matter changes can easily be assessed, but the analysis of cortical complexity seems to have been broadly neglected for this age interval.

**Methods:**

Magnetic resonance imaging (MRI) was used to acquire structural brain images. The study involved 17 adolescents (mean age 14.1 ± 0.27, 11 girls) who were compared with 14 young adults (mean age 24.24 ± 2.76, 7 women) for measures of brain complexity (fractal dimension - FD), grey matter (GM) volume and surface-area of cortical ribbon. FD was calculated using box-counting and Minkowski-Bouligand methods; FD and GM volume were measured for the whole brain, each hemisphere and lobes: frontal, occipital, parietal and temporal.

**Results:**

The results show that the adults have a lower cortical complexity than the adolescents, which was significant for whole brain, left and right hemisphere, frontal and parietal lobes for both genders; and only for males in left temporal lobe. The GM volume was smaller in men than in boys for almost all measurements, and smaller in women than in girls just for right parietal lobe. A significant Pearson correlation was found between FD and GM volume for whole brain and each hemisphere in both genders. The decrease of the GM surface-area was significant in post-adolescence for males, not for females.

**Conclusions:**

During post-adolescence there are common changes in cortical complexity in the same regions for both genders, but there are also gender specific changes in some cortical areas. The sex differences from different cortical measurements (FD, GM volume and surface-area of cortical ribbon) could suggest a maturation delay in specific brain regions for each gender in relation to the other and might be explained through the functional role of the corresponding regions reflected in gender difference of developed abilities.

## Background

Morphological changes in the brain occur throughout life span [1, 2], involving grey matter (GM) and white matter (WM) and affecting both brain structure and shape complexity [3–6]. From post-mortem studies it is known that cellular changes, as regionally variable synaptic pruning [7] together with increased myelination of intra-cortical fibres [8] continue during childhood and adolescence, and that this may underlie structural changes observable at MRI resolution [1]. The total GM volume peaks during childhood/adolescence and decreases immediately afterwards [2]. Sowell et al. [9, 10] also reports a significant reduction of GM density from childhood to adolescence and from adolescence to young adulthood. The latter is a critical period for general maturation and development, with cognitive, emotional and social implications [10]. Analyzing brain structure at various stages of development and aging provides thus clues to the variation of cognitive performance seen throughout the human life span [1, 11], and maturation and aging may be seen as two different processes that occur during the life-span, partly overlapping and without a clear demarcation between them [12].

Furthermore, GM volume variations do not only follow a complex pattern of maturation, but the peak of change differs for males and females across brain lobes [13]. Sex differences in grey-white matter composition have been reported [14]. This dimorphism in brain structure development has been explained through physiological changes during brain development related to the action on sex steroid receptors [15–17], with adolescence being the age where the majority of steroid-dependent remodelling brain changes take place [18]. Gender differences in brain structure development were reported at the level of the whole brain, cerebral hemispheres, different lobes, and even specific regions and areas within a lobe [16, 19]. Brain morphological dimorphism is consistent with cognitive functions development e.g. better language abilities for women and better spatial performance for men [20, 21]. However, while volumetric measurements provide important quantitative information, they do not reflect changes of shape irregularities of the cortical mantle that might appear during development accompanying volumetric changes of grey matter - for the same volume the shape could be different. Similarly, surface-area measurements of the brain alone do not provide information about the shape because the surface of the brain consists of a succession of foldings described by gyri (convolutions) and sulci (fissures) which confer a variability of the cortical surface visible at the macroscopic level. The anatomical pattern of the cortex ranges in shape complexity, can be different from one region to another, and varies across individuals according to age and gender [3, 22]. A possible way to quantify the changes of the cortical sheet is the fractal dimension (FD), which measures the shape complexity quantifying the spatial frequency of the “irregularities/details” of the cortical shape into a single numeric value [23]. FD may be seen as an estimation of gyrification, through a combination of sulcal depth, the frequency of folding, and the convolution of gyral shape [24]. The higher the irregularities and details of cortical surface, the higher the FD values. Being a natural fractal, an individual brain or parts of it can be characterized by their own fractal dimension and allow comparisons between subjects and groups in different stages of development and aging, in the absence of disease or in pathological conditions.

Wu et al. have computed FD during fetal life [25] and shown that the increasing values of FD are positively correlated to the gestational age, and particularly after 28 weeks of gestation the value of FD increases more rapidly because of faster development of convolved folds. In the same study a comparison was done between a group with cortical dysplasia and a matched control group, the low FD found in the first group showing that developing delay might mean less cortical complexity. Another study was done by Esteban et al. for infants at 12 months of age where a significant decrease of the FD was found in a group of infants with intrauterine growth restriction when compared with preterm or at-term controls suggesting higher vulnerability on the developing brain in the first group [26]. Blanton et al. [3] found an increasing fractal complexity in frontal lobe regions when analysing children between 6 and 16 years old. This finding may reflect subtle reorganization of sulcal topography with increases in secondary branching in the frontal lobe with age, myelinisation and synaptic remodelling that continue in the second decade of life being most likely responsible for these modifications [3]. In adults, cortical complexity measured by FD is also positively correlated with the number of years of education and the intelligence quotient [24, 27]. The FD declines in elderly people as gyral crowns became narrower and sharper and sulci became flattened and opened up [28–30].

However, the period between adolescence and adulthood seems to be overlooked for FD measurements. The volumetric measurement differences from post-adolescence should raise interest into investigating their “counterpart” in structural description, namely the cortical complexity.

The purpose of the present study is to investigate the applicability of cortical complexity methods in the post-adolescence period according to gender. The methods used for the calculation of FD were already published [31], but a more regional calculation of FD is done such as for individual lobes, left (LH) and right (RH) hemispheres, and for the whole brain (WB). The FD methods were applied on GM structure (cortical ribbon) similarly to the paper by King et al. [32]. A measurement of GM volume was also done for all regions mentioned and the measurement of the surface-area for the cortical ribbon was done for WB and each hemisphere separately.

## Methods

### Subjects

17 adolescents, 11 girls and 6 boys, (mean age 14.1 ± 0.27 years, range 13.3-14.4) were compared with 14 adults, 7 women and 7 men (mean age 24.24 ± 2.76 years, range 21–30.1). When the groups were divided according to gender, the means were as follows: girls: 14.13 ± 0.13 years, range 14–14.3, boys: 14.05 ± 0.45 years, range 13.3-14.4, women: 22.66 ± 1.08 years, range 21–23.8, men: 25.83 ± 3.08 years, range 22.2-30.1. The adolescent group came from a south-west Norwegian municipality and the adult group were students at University of Bergen, Norway. For all subjects their native language is Norwegian and all of them were still in the educational system. Estimated full IQ was obtained with a short-form of the standardized Norwegian version of the Wechsler Intelligence Scales for Children (WISC-III) [33]. The mean IQ values were as follows: girls: 98.27 ± 6.13, boys: 109.50 ± 19.38. For the adult group no IQ estimation was done.

The subjects from both groups were selected having as main criterion “no learning disorders” since they were initially recruited as control groups for different dyslexia studies. The adolescent group comes from a common pool of subjects used as control group in previous publications [34–37] and their corresponding MR images which met the technical criteria to be processed in FreeSurfer were selected. The adult group was planned to serve as control group for another dyslexia study which was not finalized. A further motivation was a study by Sowell et al. [9] who found post-adolescence changes comparing groups of adolescents and adults of ages similar to subjects in this paper, allowing comparison with their results.

Written informed consent was obtained from all subjects and, in the case of adolescents, it was also obtained from their parents. The subjects were also required to sign an informed consent letter about health status disclosure. Prior to the scanning, the subjects and, in the case of adolescents, their parents were interviewed by an MR technician about health status, including routine questions associated with MR safety screening. The study was approved by the Regional Ethics Committee for Medical Research (REK-Vest).

### Image acquisition

MR scanning of the subjects was performed on a 1.5 T Siemens Vision Plus scanner (Siemens AG, Erlangen, Germany) equipped with 25 mT/m gradients, using a standard head coil. A T1-weighted MPRAGE pulse sequence was used with the following parameters: FA/TR/TE/FOV/matrix = 10°/9.7 ms/4 ms/256 mm × 256 mm/192 × 256. Slice thickness: 1.0 mm. Voxel size 1.0 × 1.0 × 1.0 mm.

### Image processing

The volumetric analysis, using the FreeSurfer software package (version 4.5.0) [38–40] was based on two consecutive T1-volumes acquired during a single examination. Skull stripping [38], intensity normalization, and Talairach conversion [39] was performed on FreeSurfer-specific volumes (*mgz) converted from DICOM images. Then, the volumes were averaged to improve signal-to-noise ratio and obtain a better representation of the volume. The automated procedures for volumetric measurements of the cortical mantle are described in [40–44].

In the following step, a subcortical segmentation was performed [44, 45]. The segmentation produces volumes for a number of subcortical structures. Using training data, the procedure classifies each voxel of the image as belonging to a subcortical region found automatically from manual labeling of a training set [46, 47]. This classification is based on the voxel’s location in the volume, the neighbouring voxels’ tissue classes, and the intensity value in each voxel. It has been shown that this automatic labeling procedure is comparable in accuracy to manual labeling [44]. In addition, the volumetric method used in FreeSurfer obtained good results compared to other automated methods for the calculation of the GM for brain image [48].

**Figure 1:**
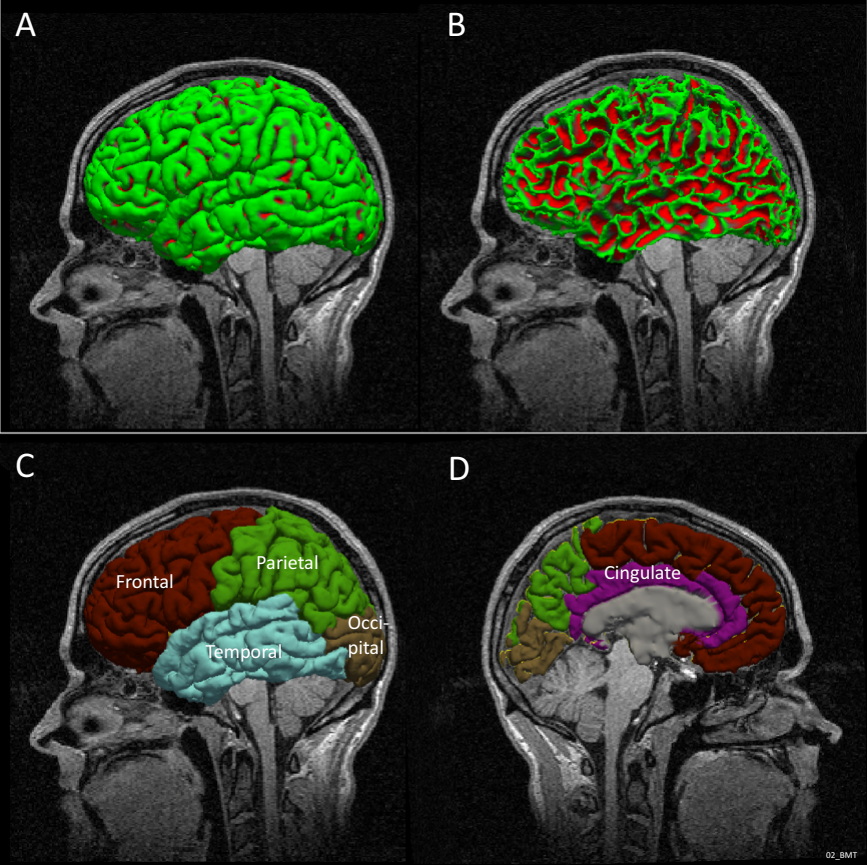
**Lateral visualisation of the pial surface in left hemisphere in one of the subjects (A) and white matter surface in the same subject and position (B). Visualization of the cortical parcellation: external lateral view (C) and mid-sagittal section (D)** **extracted from FreeSurfer.**

**Figure 2:**
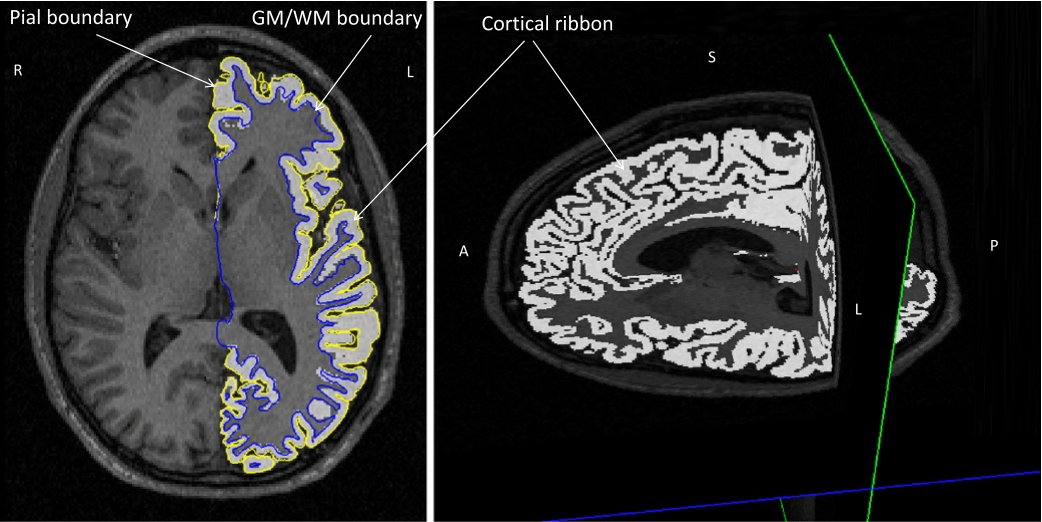
**Illustration of the grey matter structure - cortical ribbon delimitated by the pial boundary on the outside and the grey-white boundary on the inside in all three sections: transversal, sagittal and coronal.**

For all subjects, it was necessary to control the results after both the surface reconstruction and the subcortical segmentation processes due to differences in image quality and biological variability. We corrected the errors in the surface reconstruction and in the subcortical labeling using the methods described in [45]. Volumes were then re-processed. Lobes were defined according to Freesurfer convention (Figure 1). To extract the surface of the grey matter structure, delimitated by the pial border on the outside and the grey-white matter border on the inside (Figure 2), expressed in voxels, a code in Matlab R2012a (MathWorks, Natick, MA, USA) was used.

### Calculation of the fractal dimension

For the calculation of the FD for the segmented GM structure the box-counting (BC) and Minkowski-Bouligand (MB) methods were used. The analyses were based on in-house developed software written in Matlab R2012a. The details of the methods have been described in a previous study [31].

### Box-counting (BC) method

**Figure 3:**
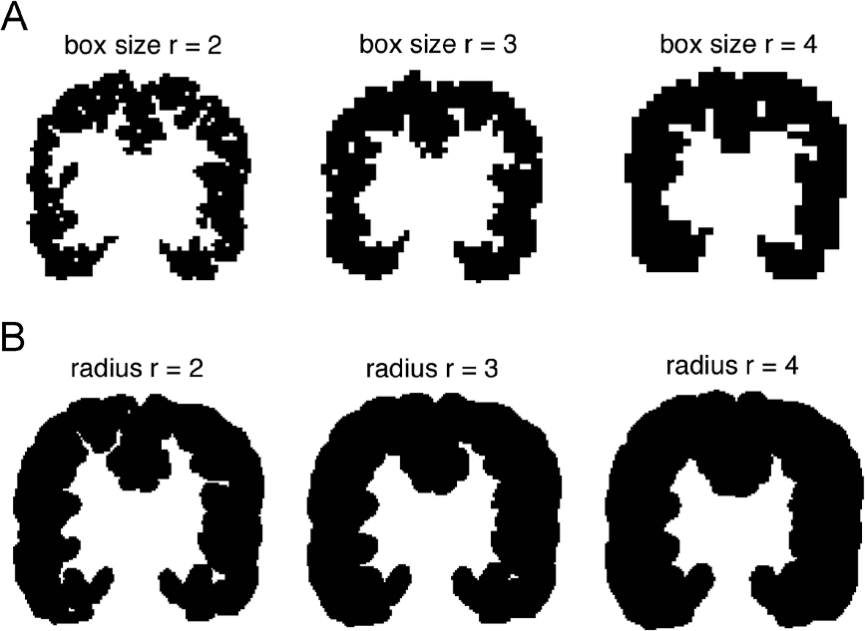
**Illustration of the methods on the cortical ribbon (A) the box-counting method and (B) Minkowski-Bouligand method.** Successive images of the cortical ribbon covered with boxes with increasing size, respectively increasing “dilatations”. The illustration is done on the middle coronal slice extracted after the construction of the boxes/dilatations on the three dimensional brain.



The GM structure (GM ribbon) is covered with 3D boxes in the BC method, which are arranged in a regular lattice and the boxes containing the GM are counted (Figure 3A). The number of boxes ***(N)*** needed to cover the whole structure varies with the linear size ***(r)*** of the box as ***N*** *~* ***r***^***-D***^, where ***D*** is the fractal dimension.

For non-Euclidean objects, ***D*** is a non-integer number. This refers to the fine structure of the fractals, i.e. by decreasing the size of the ruler one covers more details. For the results presented in the present study, the edge length of the boxes was increased by one voxel per iteration, within the range from ***r = 2*** to ***r = 30*** voxels for whole brain and hemispheres and within the range ***r = 2*** and ***r = 14*** for lobes.

### Minkowski-Bouligand (MB) method

For the MB method, the studied structure is covered with spheres of radius ***r*** (Figure 3B), resulting in a dilatation ***V(r)*** of the original object. The fractal dimension ***D*** for an object embedded in a 3 dimensional space is given by the function:


In the study, for each cycle of the procedure, the linear size ***r*** was increased by one voxel per iteration, within the range from ***r = 1*** to ***r = 7*** voxels.

## Results

The results show FD measurements using two different methods, BC and MB method and the measurements of GM volume. Each measure was computed for the whole brain, each hemisphere, as well as for lobes.

The comparisons were done between the adolescent and the adult group according to gender (i.e. boys with men and girls with women). The comparisons were based on nonparametric tests - Mann Whitney Test using SPSS Statistics 20 package.

### FD measurements

The between-group analysis showed that the adults have significantly lower FD than the adolescents. This was true in both genders for the whole brain, both hemispheres, and frontal and parietal lobes bilaterally. Findings were reproduced locally in the left temporal lobe for males only (Table 1). The results for BC and MB methods are similar, lending confidence to the methodological approach (Table 1).

**Table 1:** **Post-adolescent changes in cortical complexity**

Comparison between adolescence and adulthood cortical complexity for each gender separately using two different methods						
	**Box-counting method**			**Minkowski-Bouligand method**		
**Region**	**girls n = 11 mean ± sd**	**women n = 7 mean ± sd**	**girls > women Mann Whitney Test (p; U)**	**girls n = 11 mean ± sd**	**women n = 7 mean ± sd**	**girls > women Mann Whitney Test (p; U)**
**Frontal LH**	**2.460 ± 0.019**	**2.439 ± 0.016**	**p = 0.013; U = 11**	**2.561 ± 0.014**	**2.537 ± 0.020**	**p = 0.013; U = 11**
**Parietal LH**	**2.421 ± 0.019**	**2.384 ± 0.017**	**p = 0.002; U = 4**	**2.530 ± 0.011**	**2.499 ± 0.019**	**p = 0.002; U = 4**
Temporal LH	2.405 ± 0.023	2.395 ± 0.017	p = 0.189; U = 24	2.525 ± 0.015	2.514 ± 0.014	p = 0.094; U = 20
Occipital LH	2.340 ± 0.023	2.322 ± 0.020	p = 0.221; U = 25	2.459 ± 0.021	2.443 ± 0.016	p = 0.052; U = 17
**Frontal RH**	**2.460 ± 0.012**	**2.427 ± 0.025**	**p = 0.013; U = 11**	**2.565 ± 0.013**	**2.539 ± 0.020**	**p = 0.01; U = 10**
**Parietal RH**	**2.419 ± 0.018**	**2.371 ± 0.036**	**p = 0.016; U = 12**	**2.540 ± 0.013**	**2.498 ± 0.025**	**p = 0.002; U = 5**
Temporal RH	2.398 ± 0.013	2.381 ± 0.022	p = 0.077; U = 19	2.529 ± 0.011	2.516 ± 0.016	p = 0.063; U = 18
Occipital RH	2.320 ± 0.027	2.317 ± 0.049	p = 0.892; U = 37	2.464 ± 0.022	2.446 ± 0.034	p = 0.298; U = 27
**LH**	2.506 ± 0.013	2.497 ± 0.016	p = 0.298; U = 27	**2.624 ± 0.013**	**2.600 ± 0.018**	**p = 0.006; U = 8**
**RH**	**2.517 ± 0.014**	**2.489 ± 0.016**	**p = 0.004; U = 7**	**2.627 ± 0.011**	**2.600 ± 0.019**	**p = 0.006; U = 8**
**WB**	**2.540 ± 0.017**	**2.513 ± 0.022**	**p = 0.016; U = 12**	**2.664 ± 0.012**	**2.637 ± 0.019**	**p = 0.003; U = 7**
	**Box-counting method**			**Minkowski-Bouligand method**		
**Region**	**boys n = 6 mean ± sd**	**men n = 7 mean ± sd**	**boys > men Mann Whitney Test (p; U)**	**boys n = 6 mean ± sd**	**men n = 7 mean ± sd**	**boys > men Mann Whitney Test (p; U)**
**Frontal LH**	**2.450 ± 0.018**	**2.417 ± 0.018**	**p = 0.004; U = 1**	**2.551 ± 0.012**	**2.522 ± 0.016**	**p = 0.004; U = 1**
**Parietal LH**	**2.402 ± 0.024**	**2.356 ± 0.033**	**p = 0.015; U = 4**	**2.517 ± 0.017**	**2.470 ± 0.026**	**p = 0.007; U = 2**
**Temporal LH**	**2.413 ± 0.019**	**2.372 ± 0.037**	**p = 0.032; U = 6**	**2.535 ± 0.009**	**2.490 ± 0.030**	**p = 0.015; U = 4**
Occipital LH	2.329 ± 0.011	2.317 ± 0.012	p = 0.116; U = 10	2.450 ± 0.012	2.427 ± 0.018	**p = 0.015; U = 4**
**Frontal RH**	**2.449 ± 0.012**	**2.404 ± 0.026**	**p = 0.010; U = 3**	**2.553 ± 0.010**	**2.520 ± 0.020**	**p = 0.004; U = 1**
**Parietal RH**	**2.397 ± 0.024**	**2.367 ± 0.025**	**p = 0.022; U = 5**	**2.514 ± 0.018**	**2.480 ± 0.024**	**p = 0.015; U = 4**
Temporal RH	2.389 ± 0.021	2.369 ± 0.024	p = 0.116; U = 10	2.519 ± 0.018	2.496 ± 0.024	p = 0.063; U = 8
Occipital RH	2.318 ± 0.037	2.317 ± 0.018	p = 0.668; U = 18	2.446 ± 0.024	2.438 ± 0.015	p = 0.568; U = 17
**LH**	2.504 ± 0.017	2.480 ± 0.023	p = 0.063; U = 8	**2.614 ± 0.010**	**2.580 ± 0.018**	**p = 0.004; U = 1**
**RH**	**2.510 ± 0.014**	**2.482 ± 0.022**	**p = 0.032; U = 6**	**2.613 ± 0.015**	**2.582 ± 0.018**	**p = 0.032; U = 6**
**WB**	**2.541 ± 0.015**	**2.511 ± 0.017**	**p = 0.015; U = 4**	**2.650 ± 0.012**	**2.618 ± 0.017**	**p = 0.004; U = 1**

Post-adolescence measurements: girls versus women; boys versus men, comparisons for fractal dimension computed using box-counting and Minkowski-Bouligand methods. The significant comparisons are written in bold, where LH = left hemisphere; RH = right hemisphere and WB = whole brain.

### GM volume and surface

Volumetric analysis revealed a post-adolescent reduction in GM volume in almost all regions for males (Table 2). For women the GM reduction was found in the right parietal lobe only (Table 2). An investigation was done on the measurements of the surfaces of GM structure at the level of whole brain, left and right hemispheres. The decrease of the GM surface was significant in post-adolescence for males (whole brain: p = 0.022, U = 5; left hemisphere: p = 0.010, U = 3 and right hemisphere p = 0.032, U = 6), but not for females.

**Table 2:** **Post-adolescent changes in grey matter volumes**

Comparison between adolescence and adulthood GM volumes for each gender separately			
	**GM**		
**Region**	**girls n = 11 mean ± sd**	**women n = 7 mean ± sd**	**girls > women; Mann Whitney Test (p; U)**
Frontal LH	105819.27 **±** 10092.60	99142.43 **±** 7696.90	p = 0.269; U = 22
Parietal LH	67123.09 **±** 6141.20	62094.43 **±** 6760.26	p = 0.160; U = 23
Temporal LH	61064.27 **±** 6307.38	61638.29 **±** 5222.18	p = 0.964; U = 38
Occipital LH	29002.45 **±** 3693.50	26791.14 **±** 2067.52	p = 0. 258; U = 26
Frontal RH	108348.45 **±** 10213.77	100872.86 **±** 8993.21	p = 0.160; U = 23
**Parietal RH**	**71064.82 ± 6580.42**	**63246.29 ± 7122.82**	**p = 0.026; U = 14**
Temporal RH	60059.45 **±** 6182.36	58535.71 **±** 4608.38	p = 0.751; U = 35
Occipital RH	29268.64 **±** 3007.64	28653.86 **±** 2540.77	p = 0.892; U = 37
LH	290398.00 **±** 25273.48	276099.14 **±** 23583.36	p = 0.258; U = 26
RH	294415.55 **±** 25088.25	278559.57 **±** 25931.16	p = 0.298; U = 27
WB	561198.84 **±** 48384.66	530169.47 **±** 47531.26	p = 0.390; U = 29
**Region**	**boys n = 6 mean ± sd**	**men n = 7 mean ± sd**	**boys > men; Mann Whitney Test (p; U)**
**Frontal LH**	**114560.50 ± 10047.40**	**95270.86 ± 7701.94**	**p = 0.010; U = 3**
**Parietal LH**	**76580.17 ± 8261.57**	**64411.71 ± 8507.64**	**p = 0.032; U = 6**
**Temporal LH**	**69790.67 ± 7485.49**	**58297.86 ± 7431.34**	**p = 0.022; U = 5**
**Occipital LH**	**32486.00 ± 4057.36**	**27016.14 ± 2778.61**	**p = 0.022; U = 5**
**Frontal RH**	**116681.00 ± 11154.30**	**97360.43 ± 7697.68**	**p = 0.010; U = 3**
**Parietal RH**	**78820.83 ± 8150.86**	**65528.71 ± 8132.11**	**p = 0.022; U = 5**
**Temporal RH**	**67066.50 ± 8730.89**	**57119.14 ± 5243.98**	**p = 0.032; U = 6**
Occipital RH	33500.00 **±** 3907.30	29446.14 **±** 4103.80	p = 0.116; U = 10
**LH**	**324345.17 ± 28824.10**	**272938.71 ± 27086.43**	**p = 0.010; U = 3**
**RH**	**326150.00 ± 30526.81**	**275716.86 ± 25912.25**	**p = 0.015; U = 4**
**WB**	**625595.87 ± 57501.99**	**523328.30 ± 52695.32**	**p = 0.070; U = 2**

Post-adolescence measurements: girls versus women; boys versus men, comparisons for grey matter volumes measured in mm^3^. The significant comparisons are written in bold, where LH = left hemisphere; RH = right hemisphere and WB = whole brain.

### Correlations

Correlations were found between GM volume and FD in several regions, for example for whole brain and each hemisphere separately (Pearson R = 0.499 - 0.864, p = <0.001-0.039) for both genders (Table 3). No Pearson correlation was found between FD and GM surface at the level of whole brain, left and right hemispheres for males or females.

**Table 3:** **Correlations between grey matter volume and cortical complexity**

Pearson Correlation, Significance (two-tailed)						
Region measurements	Female (n = 18)			Male (n = 13)		
	FD-LH	FD-RH	FD-WB	FD-LH	FD-RH	FD-WB
**GM-LH**	**.697**			**.864**		
	**.001**	**-**	**-**	**<0.001**	**-**	**-**
**GM-RH**		**.499**			**.768**	
	**-**	**.035**	**-**	**-**	**.039**	**-**
**GM-WB**			**.551**			**.810**
	**-**	**-**	**.018**	**-**	**-**	**.001**

Correlations between grey matter volume and fractal dimension for females, respectively males for the following regions: GM-LH = grey matter volume of left hemisphere; GM-RH = grey matter volume of right hemisphere; GM-WB = grey matter volume of whole brain; FD-LH = fractal dimension of left hemisphere; FD-RH = fractal dimension of right hemisphere; FD-WB = fractal dimension of whole brain. The significant values are written in bold.

## Discussion

The current study shows the applicability, as a proof of concept, of a novel method of analysis, complementary to the classical volume and surface measurements. Summarizing the main findings, the adult group has lower FD values than the adolescent group for frontal and parietal lobes in both genders (Table 1). Using two different methods to compute fractal dimension and obtaining almost the same results shows once more that the measure of cortical complexity is accurate and sensitive in detecting age related GM structural changes. The reduction of FD values is correlated to reduced GM volumes, especially in males. The reduction of GM surface-area was significant only in the male case.

A similar reduction of the FD value with age has also been found by Zhang et al. [5, 29] and Lee et al. [28] but in another age group, where the comparison was made between adults and elderly people. Here, the fractal complexity was reduced with age as the surface of the brain becomes smoother and the sulci become wider and less curved with increasing age [30]. It is, however, an open question whether the FD effects seen in adults are a continuation of the reduction of FD that already begins in adolescence or if it represents a discontinuity with the involvement of different mechanisms. It is not clear the precise moment when development (e.g. pruning or activity-dependent changes) turns into a degenerative process. On the other hand, Kalmanti and Maris [4] proposed that childhood and adolescence are the most significant brain remodelling periods throughout life, analysing 2D fractal dimension from a series of parasagittal slices. The structural complexity of the brain during development between the age 6 and 16 years revealed an accentuated increase in frontal regions in both hemispheres [3]*.* While the fractal complexity of the brain between adolescence and adulthood seems not to have been the topic of previous studies, Sowell et al. [10] reports post-adolescent GM density loss in many regions of the brain, registering as one of the biggest losses during the life span. Certain regions are expected to be largely mature by adolescence such as the lateral temporal lobes, which are involved in auditory and speech processing, and the parietal association cortices involved in spatial orientation processing and sensory functions [10]. A more precise time for the maturation of these regions should also implicate gender as a factor [15].

In addition to the FD reduction in frontal and parietal lobes for both genders, there is a particular difference for males in the left temporal lobe, where FD also decreased significantly between adolescence and adulthood. Assuming that the majority of brain regions where the FD is not significantly modified have largely reached the maturation, significant local FD differences could suggest a gender-dependent latency in maturation for the respective region. The remodelling of the cortical architecture in the left temporal lobe could be associated with the improvement of speech abilities [49]. The function of the left temporal lobe is not limited to speech perception, it also includes complex functions of language abilities (eg. comprehension and verbal memory) which might develop less or with latency in males compared to females [19–21].

No significant differences in complexity were found in occipital lobes for both genders. There are also other regions in each gender where no significant changes were detected, as already discussed above, suggesting that these regions are less anatomo-structural variable during post-adolescence and that their corresponding functions are developed by adolescence or earlier [3].

The reduction of GM is supported by previous findings [1, 10, 14], and may indicate a global effect on brain maturation and development with a decrease in GM volume from adolescence to adulthood. The reduction of the GM volume in this age interval is significant in almost all measurements for males, but just in one measurement - right parietal lobe- for females. The average age difference at puberty is approximately 1 to 2 years, earlier in females than males, and the GM volume peak occurs with a similar age difference, earlier for females [14, 17]. Based on this difference we can suppose that the GM volumes corresponding to brain maturation might be attained in females before the analysed interval in our study (14.1- 24.24 years old). However, the significant reduction of GM in right parietal in females suggests that there are still brain regions and corresponding cognitive functions in process of maturation in females in this interval. Behind this volumetric finding could be the functional aspect, noticed by Christakou et al. [50], who found more mature parietal activation patterns, even more in right hemisphere, in males than in females. The activation patterns were recorded during cognitive tasks of visuospatial information processing in adolescent and adult subjects (13 and 38 years old, respectively) [50].

### Limitations

The variability among subjects for height, weight, gender and even age is a well-established fact and reflected in different head size [51]. The correction of the cortical brain volumes according to head size/total intracranial volume is pertinent for volumetric comparison between subjects and groups, but in our study the comparisons were done for volumes uncorrected for the head size. However, at least in theory, the FD should not be related to volume [30]. Another potential confounder in the results is the lack of IQ measurements for the adult sample especially since the student population is likely to have a higher IQ than the slightly-above-average-IQ adolescent group and thus it could introduce a bias in the group differences. The cross sectional nature of the study is another limitation, a longitudinal study with a larger number of subjects could consolidate our findings. Another limitation could be the “partial volume” effect, a technical aspect that might appear in MRI on T1-weighted images, inducing an apparent loss of cortical GM as a result of increased degree of myelinisation of intra-cortical fibers [13, 16] from birth to adulthood [8]. The fact that subjects in the current study are in the sensitive age interval when this effect occurs, could influence the results.

## Conclusion

The present findings demonstrate that during post adolescence there are changes in cortical complexity in both genders. Specific sex differences in brain complexity for this age interval could suggest a delayed maturation in left temporal lobe for males. This difference might be explained through the functional role of the corresponding regions reflected in developed cognitive abilities. The measurement of FD, volumes and surfaces can contribute to a battery of tests capable of estimating the degree of brain maturation and implicit age-specific cognitive development.

## Abbreviations

MRI: Magnetic resonance imaging
FD: Fractal dimension
GM: Grey matter
WM: White matter
LH: Left hemisphere
RH: Right hemisphere
WB: Whole brain
REK-vest: Regional Ethics Committee for Medical Research (translated in English)
MPRAGE: Magnetization-prepared rapid acquisition with gradient echo
FA: Flip angle
TR: Repetition time
TE: Echo time
FOV: Field of view
DICOM: Digital imaging and communications in medicine
BC: Box-counting method
MB: Minkowski-Bouligand method.
